# *SmGA2ox4* plays a positive role in improving the salt tolerance and tanshinone accumulation of *Salvia miltiorrhiza*

**DOI:** 10.1093/hr/uhag058

**Published:** 2026-02-26

**Authors:** Sijia Zeng, Yifan Li, Shiying Wang, Yihua Liang, Zhijing Yu, Zisong Yang, Pengda Ma, Jingying Liu

**Affiliations:** College of Life Sciences, Northwest A&F University, Yangling, Shaanxi 712100, China; College of Life Sciences, Northwest A&F University, Yangling, Shaanxi 712100, China; College of Resources and Environment, ABA Teachers College, Wenchuan 623002, China; College of Life Sciences, Northwest A&F University, Yangling, Shaanxi 712100, China; Jilin Provincial Key Laboratory of Agricultural Biotechnology, Jilin Academy of Agricultural Sciences (Northeast Agricultural Research Center of China), Changchun 130033, China; College of Resources and Environment, ABA Teachers College, Wenchuan 623002, China; College of Life Sciences, Northwest A&F University, Yangling, Shaanxi 712100, China; College of Life Sciences, Northwest A&F University, Yangling, Shaanxi 712100, China

## Abstract

*Salvia miltiorrhiza,* a medicinal plant of high value, faces significant yield and quality losses due to salt stress. Identifying salt tolerance genes is therefore essential for breeding resilient varieties. Gibberellin (GA) metabolism and signaling are modulated by diverse factors, with GA 2-oxidase (GA2ox) playing a key role in stress adaptation by inactivating GA and fine-tuning growth under adverse conditions. In this work, we identified 12 GA2ox genes in *S. miltiorrhiza* and generated an *SmGA2ox4* transgenic line. Heterologous expression in *Arabidopsis thaliana* improved salt tolerance through enhanced germination, root growth, antioxidant activity, and stress-related physiological markers. Similar results were observed in transgenic hairy roots of *S. miltiorrhiza*. High-performance liquid chromatography (HPLC) analysis further showed that *SmGA2ox4* overexpression promoted tanshinone accumulation but suppressed salvianolic acid biosynthesis, whereas RNA interference (RNAi)-mediated silencing had the opposite effect. Thus, *SmGA2ox4* acts as a dual-function regulator, enhancing both salt tolerance and tanshinone production. This study establishes a novel link between GA2ox-mediated stress response and secondary metabolism in *S. miltiorrhiza*, providing a basis for engineering stress-resistant, high-quality varieties.

## Introduction

*Salvia miltiorrhiza,* a perennial herb of the genus *Salvia (Lamiaceae)*, is valued for the pharmacological activities of its dried roots and rhizomes. The herb’s bioactivity is primarily attributed to two major groups of compounds: lipophilic tanshinones and hydrophilic phenolic acids [1–3].

*Salvia miltiorrhiza* is widely distributed across numerous provinces in China, including Henan, Hebei, Shandong, Shanxi, and Shaanxi. These regions are primarily characterized by semiarid hilly terrain, where soil salinization poses a significant constraint on plant growth. Under high-salinity conditions, *S. miltiorrhiza* displays pronounced growth inhibition as a physiological adaptation. To mitigate salt stress, the plant employs protective strategies such as the accumulation of osmoprotectants and the regulation of antioxidant enzyme activities [4]. These coordinated physiological and biochemical adjustments collectively enhance the salt stress tolerance of *S. miltiorrhiza*.

Plant secondary metabolites are indispensable for adaptation to environmental changes. In *S. miltiorrhiza*, moderate salt stress enhances the biosynthesis of total tanshinones, whereas excessively high salinity suppresses both tanshinone accumulation and plant growth. Further investigations indicate that under mild salt stress, *S. miltiorrhiza* accumulates secondary metabolites such as tanshinones and salvianolic acids to facilitate adaptation. Moreover, the expression of multiple key enzyme genes and transcription factors involved in the biosynthesis of tanshinones and salvianolic acids is modulated in response to abiotic stress. For instance, the transcription factor *SmMYC2*, a positive regulator of tanshinone biosynthesis, is upregulated under salt stress, thereby enhancing plant salt tolerance [5]. Similarly, the upregulation of MYB transcription factors *SmMYB13* and *SmMYB70* not only confers responsiveness to salt stress but also elevates the content of total flavonoids and salvianolic acids in *S. miltiorrhiza* cells [6]. These findings offer critical insights for elucidating the molecular regulatory mechanisms underlying the salt stress response in *S. miltiorrhiza*.

As a major plant hormone, gibberellin (GA) has long been a central and widely studied topic in plant science globally. Plant dwarfism is a typical phenotypic outcome of impaired gibberellin signaling. Leveraging this mechanism, the gibberellin signaling pathway has been extensively utilized in breeding programs to develop lodging-resistant crop varieties, marking the advent of the first ‘Green Revolution’ in agriculture. To adapt to dynamic environmental conditions, plants require the capacity for precise and rapid modulation of their gibberellin (GA) content. Similar to other phytohormones, GA homeostasis is largely maintained through enzymatic inactivation. Multiple GA inactivation mechanisms have been identified across species. For example, a 16,17-dihydrodiol-mediated inactivation pathway was revealed in rice mutants [7]. In *Arabidopsis thaliana*, the SABATH family methyltransferases GAMT1 and GAMT2 catalyze the carboxyl methylation of GAs at the C-7 position [8]. Nevertheless, the most prevalent inactivation mechanism is 2β-hydroxylation, which is primarily catalyzed by GA 2-oxidases (GA2oxs).

GA 2-oxidases (GA2oxs) belong to the 2-oxoglutarate-dependent dioxygenase (2OGD) superfamily and are encoded by multiple genes. These enzymes primarily function as 2β-hydroxylases, catalyzing the hydroxylation of active GAs and further oxidizing 2β-hydroxy groups to ketones [9, 10]. Most GA2oxs utilize C_19_-GAs as substrates, hydroxylating GA₁ and GA₄ as well as their precursors G_9_ and GA₂₀ to generate inactive forms such as GA_8_, GA₃₄, GA_51_, and GA_29_ [10]. A distinct class of GA2oxs, including *AtGA2ox7* and *AtGA2ox8* in *A. thaliana*, preferentially inactivates C₂₀-GAs. Heterologous expression of these genes leads to dwarf phenotypes in both *A. thaliana* and tobacco [11]. Furthermore, recombinant *SoGA2ox1* from spinach demonstrates dual substrate specificity, acting on both C_19_- and C₂₀-GAs [12]. The GA2ox gene family is widely distributed in higher plants. By the late 1990s and early 2000s, GA2ox homologs had been identified in several species, including *A. thaliana* [13–15], pea [16, 17], spinach [12], poplar ,[18], and rice [19, 20].

Among the complex responses of plants to environmental stresses, growth retardation serves as a conserved adaptive strategy that facilitates the reallocation of resources toward defense-related processes [21, 22]. In potato, transgenic lines overexpressing *StGA2ox1* displayed elevated chlorophyll content, relative leaf water content, and free proline levels under drought stress, resulting in enhanced drought tolerance. The expression of *StGA2ox2*, *StGA2ox4*, *StGA2ox8*, *StGA2ox9*, and *StGA2ox10* was also significantly induced by low-temperature stress [23]. In *A. thaliana*, *AtGA2ox6* expression is strongly upregulated by salt stress specifically in root tip tissues, where it modulates the expression of cell cycle-related genes and inhibits root elongation. The *AtGA2ox7* gene is likewise markedly induced under high salinity, contributing to salt stress resistance and associated morphological adaptations [24]. Furthermore, overexpression of *GhGA2ox1* in upland cotton enhances tolerance to both drought and salt stress, accompanied by increased leaf relative water content, as well as elevated levels of free proline and chlorophyll [25]. Despite the established roles of GA2ox enzymes in stress adaptation and growth regulation across various plant species, their specific functions in the medicinal plant *S. miltiorrhiza*, particularly concerning abiotic stress tolerance and the biosynthesis of bioactive compounds like tanshinones and salvianolic acids, have not been elucidated.

In this study, we systematically identified 12 GA2ox genes in *S. miltiorrhiza* and characterized their gene structures, conserved motifs, and promoter *cis*-acting elements. Phylogenetic analysis was performed to classify these genes and infer their evolutionary relationships. Based on transcriptome data indicating the strong responsiveness of *SmGA2ox4* to jasmonic acid (JA) induction, we selected this gene for functional investigation and generated transgenic lines for subsequent analysis. Our findings demonstrate that heterologous expression of *SmGA2ox4* in *A. thaliana* significantly enhances salt stress tolerance. Furthermore, functional validation in *S. miltiorrhiza* hairy roots confirmed that *SmGA2ox4* also mediates salt stress adaptation in its native host. Additionally, *SmGA2ox4* was found to regulate the accumulation of key medicinal compounds in *S. miltiorrhiza*. Collectively, this study provides important insights for genetically enhancing salt stress resistance and modulating the production of valuable bioactive compounds in *S. miltiorrhiza*.

## Results

### Genome-wide identification and characterization of GA2ox genes in *S. miltiorrhiza*

Through homology analysis of GA2ox amino acid sequences from *A. thaliana* and *S. miltiorrhiza*, we identified 12 nonredundant GA2ox enzymes in the *S. miltiorrhiza* genome. Table 1 summarizes the characteristic parameters of these putative GA2ox members, including CDS length, protein length, theoretical molecular weight, isoelectric point (pI), and predicted subcellular localization. The CDS lengths of the 12 predicted SmGA2ox genes range from 408 to 1383 bp, encoding proteins comprising 135–460 amino acid residues, with molecular weights ranging from 14.55 to 51.43 kDa and pI values between 4.90 and 7.91. Subcellular localization predictions indicated that all SmGA2ox proteins are localized to the cytoplasm (Table S1).

**Table 1:** Characteristics of GA2ox gene family members in *S. miltiorrhiza*

Gene name	Gene identifier	CDS length (bp)	Protein length (aa)	Mw molecular weight (Da)	Theoretical pI	Instability index	Subcellular localization
*SmGA2ox1*	*SMil_00005648*	939	312	34 681.59	6.31	46.39	Cytoplasm
*SmGA2ox2*	*SMil_00028460*	936	311	34 351.41	5.85	50.53	Cytoplasm
*SmGA2ox3*	*SMil_00008040*	594	197	22 031.09	6.19	45.53	Cytoplasm
*SmGA2ox4*	*SMil_00019079*	678	225	24 063.03	5.71	44.01	Cytoplasm
*SmGA2ox5*	*SMil_00019080*	408	135	14 548.78	5.59	19.44	Cytoplasm
*SmGA2ox6*	*SMil_00003584*	930	309	34 430.1	5.16	44.76	Cytoplasm
*SmGA2ox7*	*SMil_00007153*	1383	460	51 425.18	6.3	48.32	Cytoplasm
*SmGA2ox8*	*SMil_00014304*	594	197	22 080.15	6.12	44.15	Cytoplasm
*SmGA2ox9*	*SMil_00017774*	975	324	36 056.31	6.67	38.19	Cytoplasm
*SmGA2ox10*	*SMil_00020663*	843	280	31 247.89	6.24	30.12	Cytoplasm
*SmGA2ox11*	*SMil_00021366*	729	242	27 056.86	4.9	43.61	Cytoplasm
*SmGA2ox12*	*SMil_00022299*	849	282	31 651.15	7.91	42.59	Cytoplasm

Including CDS length, protein length, theoretical molecular weight, isoelectric point (pI), and predicted subcellular localization.

**Table S1:** 

**Figure 1:**
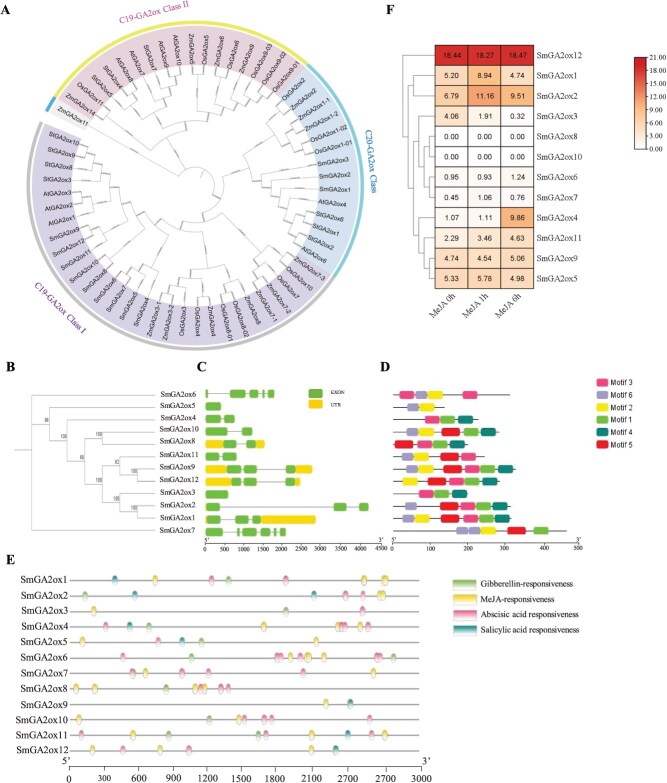
Genome-wide identification and characterization of GA2ox genes in *S. miltiorrhiza*. **(A)** Phylogenetic analysis of GA2ox proteins from *S. miltiorrhiza* and four representative plant species. The neighbor-joining tree was constructed in MEGA X with 1000 bootstrap replicates based on full-length protein sequences. Species abbreviations: *S. miltiorrhiza* (Sm), *O. sativa* (Os), *A. thaliana* (At), *Z. mays* (Zm), *S. tuberosum* (St). **(B)** Phylogenetic tree of *S. miltiorrhiza* GA2ox proteins showing evolutionary relationships among family members. **(C)** Exon–intron structures of SmGA2ox genes visualized using TBtools. **(D)** Conserved protein motifs identified by MEME analysis of full-length GA2ox sequences. Six distinct motifs are represented by different colored boxes. **(E)**  *Cis*-acting elements in SmGA2ox promoters predicted by PlantCARE. Elements associated with GA, JA, ABA, and SA signaling responses are shown. **(F)** Heatmap depicting expression patterns of 12 SmGA2ox genes following MeJA treatment.

To elucidate the evolutionary relationships of GA2ox family proteins, a phylogenetic tree was constructed using the neighbor-joining method based on the protein sequence similarities of GA2ox from *S. miltiorrhiza* (Sm), *Oryza sativa* (Os), *A. thaliana* (At), *Zea mays* (Zm), and *Solanum tuberosum* (St). The GA2ox gene families from these five species were classified into three distinct groups. The *S. miltiorrhiza* GA2ox members were exclusively distributed in two of these groups: nine SmGA2ox proteins clustered within C19-GA2ox Class I, while the remaining three grouped into C20-GA2ox Class (Fig. 1A and B).

### Gene structure and promoter analysis of *SmGA2ox* genes

Gene structure analysis performed with TBtools revealed that the number of exons in SmGA2ox genes ranges from 1 to 6, with *SmGA2ox3* and *SmGA2ox5* containing only a single exon (Fig. 1C). To further explore the functional characteristics of these genes, conserved motifs were analyzed using the MEME program. Most members exhibit a tandem arrangement of three to four motifs, though distinct variations in motif composition were observed among different members (Fig. 1D).

To investigate the potential regulatory mechanisms of SmGA2ox genes under stress conditions, we extracted their upstream promoter sequences and predicted *cis*-acting elements using the PlantCARE database. The analysis revealed that SmGA2ox promoters contain multiple hormone-responsive elements, including those associated with GA, JA, abscisic acid (ABA), and salicylic acid (SA) signaling (Fig. 1E), suggesting their potential involvement in abiotic stress responses in *S. miltiorrhiza*.

Given the well-documented role of JA in regulating salt stress responses and secondary metabolite accumulation in *S. miltiorrhiza* [26–29], we examined the expression of SmGA2ox genes under methyl jasmonate (MeJA) treatment. Transcriptome analysis showed that *SmGA2ox4* expression was significantly upregulated after 6 h of MeJA exposure (Fig. 1F). Based on this induced expression pattern, *SmGA2ox4* was selected for further functional studies to elucidate its role in salt stress tolerance and the regulation of secondary metabolism.

### Salt, MeJA, and GA₃ treatments significantly induce *SmGA2ox4* expression

**Figure 2:**
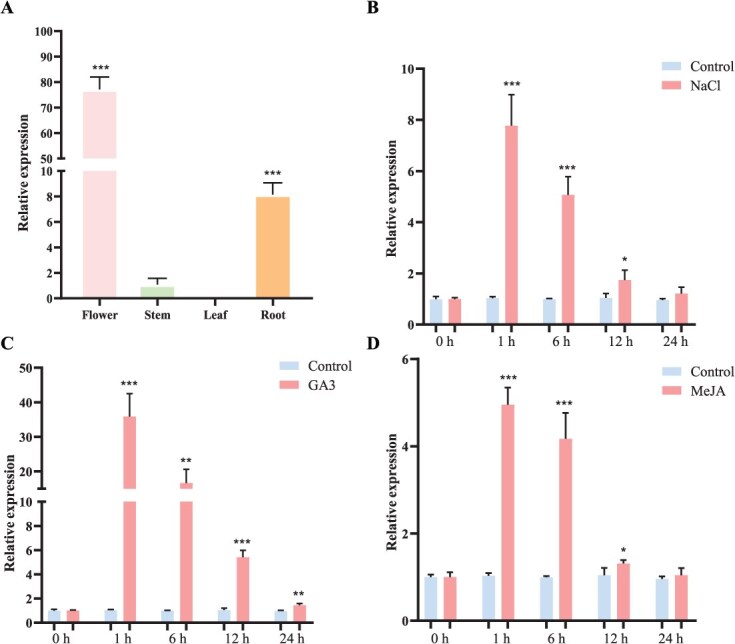
Expression patterns of *SmGA2ox4* in response to salt, GA₃, and MeJA treatments. **(A)** Tissue-specific expression profile of *SmGA2ox4* in WT *S. miltiorrhiza* as determined by RT-qPCR. Gene expression levels were normalized to Actin, with the expression level in stems set as 1. **(B–D)** Temporal expression patterns of *SmGA2ox4* in hairy roots following 24-h treatments with **(B)** 150 mM NaCl, **(C)** 100 μM GA₃, and **(D)** 100 μM MeJA. Expression levels were normalized to *SmActin* and are presented relative to the 0-h control group (set as 1). Data represent mean ± standard deviation (SD) of three biological replicates. Significant differences between treatment groups and the control were determined by Student’s *t*-test (^*^P < 0.05, ^**^P < 0.01, ^***^P < 0.001).

Real-time quantitative PCR (RT-qPCR) analysis revealed that *SmGA2ox4* exhibited high expression levels in stems, leaves, and flowers, while its expression in roots was notably low or undetectable (Fig. 2A)*.* To elucidate the biological function of *SmGA2ox4* and its potential role in salt stress tolerance, we examined its transcriptional dynamics in response to NaCl treatment. In *S. miltiorrhiza* hairy roots, NaCl treatment significantly induced *SmGA2ox4* expression, leading to an 8-fold increase within 1 h. Transcript levels subsequently declined gradually, returning to baseline 24 h after treatment (Fig. 2B).

Similarly, treatment with exogenous gibberellin (GA₃) and MeJA also markedly upregulated *SmGA2ox4* expression. Transcript levels peaked at 1 h post-treatment, reaching 26-fold and 5-fold of the control levels under GA₃ and MeJA treatments, respectively (Fig. 2C and D). These response patterns resemble the transient induction observed under salt stress, suggesting that *SmGA2ox4* expression is responsive to multiple hormonal and environmental signals.

### Overexpression of *SmGA2ox4* enhances salt stress tolerance in *A. thaliana*

To investigate the functional role of *SmGA2ox4*, we generated transgenic *A. thaliana* lines overexpressing the *SmGA2ox4* gene via the Agrobacterium-mediated floral dip method, using the pCAMBIA1300-221-myc-*SmGA2ox4* vector. Two independent overexpression lines (designated *SmGA2ox4*-OE-1 and *SmGA2ox4*-OE-4) with relatively high transgene expression levels were selected based on qPCR analysis (Fig. S1) for subsequent phenotypic characterization under salt stress conditions.

**Figure 3:**
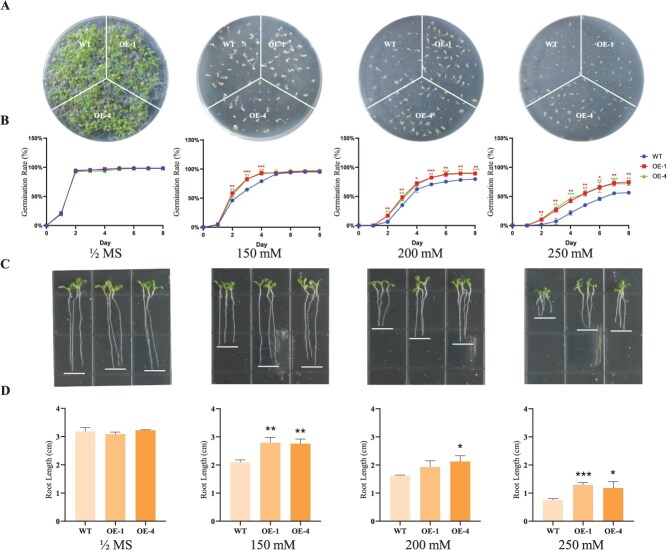
Germination rate and primary root length of transgenic *A. thaliana* seeds under salt stress. **(A and B)** Germination rates of WT and transgenic lines (*SmGA2ox4*-OE-1 and *SmGA2ox4*-OE-4) after 8 days of culture on one-half MS medium (control) and one-half MS medium supplemented with 150, 200, or 250 mM NaCl. **(C and D)** Primary root lengths of WT and transgenic lines measured after 8 days of growth under the same conditions as in (A and B). Data represent mean ± SD of three biological replicates. Significant differences between transgenic lines and WT under each treatment were determined by Student’s *t*-test (^*^P < 0.05, ^**^P < 0.01, ^***^P < 0.001).

To assess the effect of *SmGA2ox4* on salt tolerance during early development, seeds of wild-type (WT) and transgenic lines were sown on one-half MS medium supplemented with different concentrations of NaCl. Under high salt conditions (≥200 mM NaCl), the germination rates of both OE-1 and OE-4 seeds were significantly higher than those of WT. This phenotypic advantage emerged during the initial germination stage and became more pronounced throughout subsequent seedling growth (Fig. 3A and B). Compared to WT, the *SmGA2ox4* overexpression lines maintained a higher germination rate under salt stress, indicating that *SmGA2ox4* overexpression effectively alleviates NaCl-induced inhibition of seed germination and confers enhanced stress resistance during early plant development.

We further investigated the effect of *SmGA2ox4* overexpression on primary root development under salt stress conditions. On standard one-half MS medium, no significant differences in root length were observed between WT and transgenic lines. However, when grown on one-half MS medium supplemented with 150 mM or higher concentrations of NaCl, both transgenic lines exhibited significantly longer primary roots compared to WT (Fig. 3C and D). These morphological differences suggest that *SmGA2ox4* overexpression enhances root system adaptability, potentially facilitating improved water and nutrient acquisition under saline conditions.

**Figure 4:**
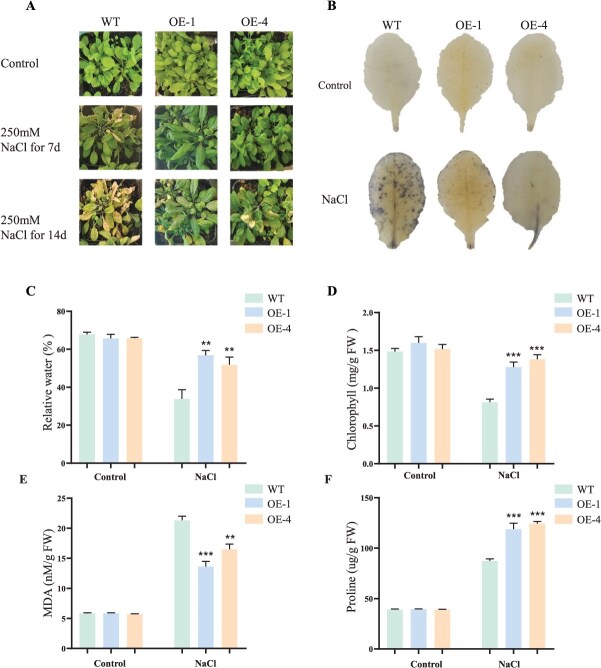
Phenotypic and physiological responses of transgenic *A. thaliana* seedlings to salt stress. **(A)** Phenotypes of WT and *SmGA2ox4*-overexpressing lines following 7 and 14 days of treatment with 250 mM NaCl. **(B)** Histochemical detection of superoxide anions (O^₂-^) and hydrogen peroxide (H₂O₂) accumulation via NBT staining after salt stress treatment. **(C–F)** Physiological parameters of WT and SmGA2ox4-overexpressing lines after salt stress treatment: **(C)** leaf RWC, **(D)** chlorophyll content, **(E)** MDA content, and **(F)** proline content. Data represent mean ± SD of three biological replicates. Significant differences between transgenic lines and WT were determined by Student’s *t*-test (^*^P < 0.05, ^**^P < 0.01, ^***^P < 0.001).

To assess the protective role of *SmGA2ox4* under prolonged salinity stress, we evaluated the growth performance of transgenic *A. thaliana* seedlings under continuous NaCl treatment. After 7 days of exposure to 250 mM NaCl, WT plants displayed noticeable stress phenotypes, including leaf yellowing and wilting. In contrast, both *SmGA2ox4*-OE-1 and *SmGA2ox4*-OE-4 transgenic lines maintained robust growth. By day 14 of stress treatment, WT plants exhibited severe growth inhibition accompanied by extensive leaf chlorosis and necrosis. Although mild stress symptoms were observed in the transgenic lines, their overall growth performance remained significantly better than that of WT plants (Fig. 4A).

Histochemical staining with nitroblue tetrazolium (NBT) revealed that under salt stress, WT plants accumulated substantially higher levels of superoxide anions and H₂O₂ compared to both *SmGA2ox4* overexpression lines (Fig. 4B).

Physiological analysis showed no significant differences between transgenic and WT plants under normal growth conditions. However, after salt stress treatment, all plants showed increases in leaf relative water content (RWC), proline, chlorophyll, and malondialdehyde (MDA) content. Specifically, the RWC of OE-1 and OE-4 reached 56.81% and 51.85%, respectively, significantly higher than the WT value of 33.86% (*P* < 0.01). Chlorophyll content in the transgenic lines (1.28 and 1.38 mg/g) was also significantly elevated compared to WT (0.82 mg/g, *P* < 0.001). Proline accumulation in OE-1 and OE-4 (118.92 and 124.46 μg/g) significantly exceeded that in WT (87.41 μg/g, *P* < 0.01). Conversely, MDA content—an indicator of membrane lipid peroxidation—was significantly lower in the transgenic lines (13.65 and 15.50 nmol/g) than in WT (21.31 nmol/g, *P* < 0.01) (Fig. 4C–F).

**Figure 5:**
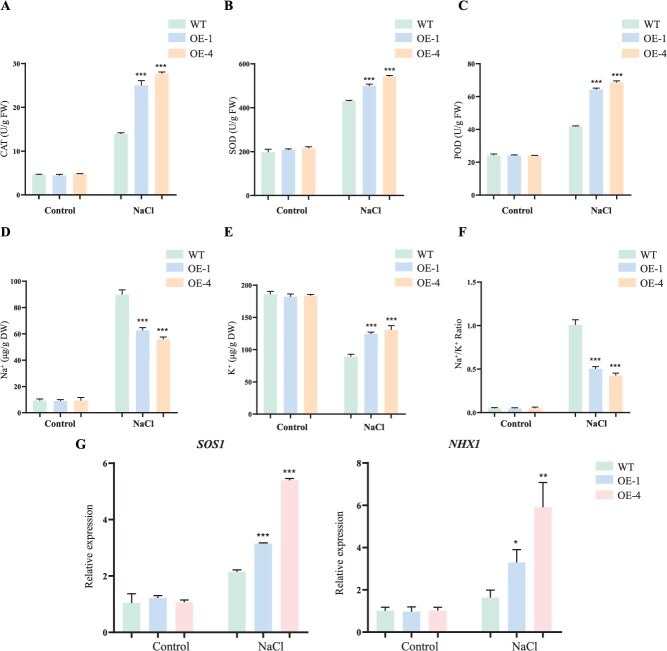
Antioxidant enzyme activities, ion contents, and expression of salt-responsive genes in transgenic *A. thaliana* under salt stress. **(A–C)** Activities of antioxidant enzymes in WT and *SmGA2ox4*-overexpressing lines after salt stress treatment: **(A)** CAT, **(B)** SOD, and **(C)** POD. **(D and E)** Ion content analysis in seedlings after salt stress: **(D)** sodium ion (Na^+^) content, **(E)** potassium ion (K^+^) content. **(F)** Na^+^/K^+^ ratio in WT and transgenic lines under salt stress. **(G)** Expression levels of salt stress-responsive genes (*AtSOS1* and *AtNHX1*) in WT and *SmGA2ox4*-overexpressing lines, as determined by qRT-PCR. *AtActin* was used as the internal reference, and expression levels in WT under normal conditions were set to 1. Data represent mean ± SD of three biological replicates. Significant differences between transgenic lines and WT were determined by Student’s *t*-test (^**^P < 0.01, ^***^P < 0.001).

We simultaneously assessed the activities of three key antioxidant enzymes—catalase (CAT), superoxide dismutase (SOD), and peroxidase (POD)—in both transgenic and WT *A. thaliana* after 14 days of treatment with 250 mM NaCl. As shown in Fig. 5A–C, the activities of CAT, SOD, and POD were significantly elevated in the SmGA2ox4-overexpressing lines OE-1 and OE-4 compared to WT (*P* < 0.001). These results indicate that the overexpression of *SmGA2ox4* enhances the antioxidant capacity, leading to a marked reduction in reactive oxygen species (ROS) accumulation and consequently mitigating oxidative damage to cellular structures such as membranes and proteins.

The sodium (Na^+^) and potassium (K^+^) ion contents and their ratio serve as critical indicators of plant salt stress tolerance. Under saline conditions, elevated soil Na^+^ levels can disrupt intracellular ion homeostasis, inhibit enzymatic activity, and impair metabolic processes. Furthermore, Na^+^ competes with K^+^ for uptake, resulting in K^+^ deficiency and compromised plant growth. Thus, the Na^+^/K^+^ ratio is widely used to evaluate salt tolerance in plants; salt-resistant genotypes typically maintain lower Na^+^/K^+^ ratios under stress, thereby minimizing Na^+^ toxicity and preserving K^+^-dependent physiological functions.

In our study, ion content analysis revealed that, under salt stress, the *SmGA2ox4*-overexpressing lines accumulated less Na^+^ and more K^+^ than WT plants, resulting in a significantly lower Na^+^/K^+^ ratio (Fig. 5D–F). These findings suggest that *SmGA2ox4* contributes to the maintenance of ion homeostasis, further supporting its role in enhancing salt tolerance.

To elucidate the molecular mechanism by which *SmGA2ox4* enhances salt tolerance, we examined the transcript levels of two key ion homeostasis regulators—*AtSOS1* (Salt Overly Sensitive 1) and *AtNHX1* (Na^+^/H^+^ Exchanger 1)—in transgenic and WT *A. thaliana* after 14 days of salt stress. As shown in Fig. 5G, both *AtSOS1* and *AtNHX1* were significantly upregulated in the *SmGA2ox4*-overexpressing lines compared to WT under salt stress conditions.

These findings suggest that *SmGA2ox4* contributes to salt stress adaptation by modulating the expression of critical genes involved in Na^+^ efflux (*AtSOS1*) and vacuolar Na^+^ sequestration (*AtNHX1*), thereby enhancing cellular ion homeostasis and overall salt tolerance in transgenic plants.

### Overexpression of *SmGA2ox4* enhances, while RNAi-mediated silencing reduces, salt stress tolerance in *S. miltiorrhiza* hairy roots

To further validate the role of *SmGA2ox4* in plant salt tolerance, we generated transgenic *S. miltiorrhiza* hairy roots with altered *SmGA2ox4* expression (Fig. S2).

**Figure 6:**
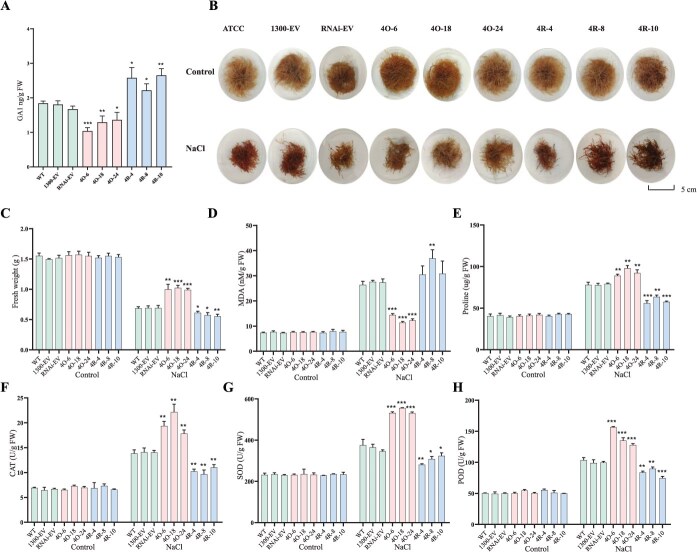
Phenotypic and physiological responses of transgenic *S. miltiorrhiza* hairy roots to salt stress. **(A)** Quantification of GA₁ content in transgenic hairy root line. **(B)** Phenotypes of hairy root lines—WT (ATCC), empty vector controls (1300-EV and RNAi-EV), SmGA2ox4-overexpressing (4O), and SmGA2ox4-RNAi-silenced (4R) lines—after 7 days of treatment with 150 mM NaCl. **(C)** FW of hairy roots under salt stress conditions. **(D and E)** Contents of MDA and proline in hairy roots following salt stress treatment. **(F–H)** Activities of antioxidant enzymes in hairy roots under salt stress: **(F)** CAT, **(G)** SOD, and **(H)** POD. Data represent mean ± SD of three biological replicates. Significant differences between transgenic lines and corresponding controls were determined by Student’s *t*-test (^*^P < 0.05, ^**^P < 0.01, ^***^P < 0.001).

First, we quantified GA₁ levels in various transgenic hairy root lines. The results clearly indicated that overexpression of *SmGA2ox4* significantly reduced GA₁ content in hairy roots (Fig. 6A), suggesting that growth restriction through modulation of gibberellin activity may contribute to enhanced environmental stress tolerance in plants.

On standard 6,7-V medium, the growth of *S. miltiorrhiza* hairy roots showed no significant differences among the WT (ATCC), empty vector controls (1300-EV and RNAi-EV), *SmGA2ox4*-overexpressing, and *SmGA2ox4*-RNA interference (RNAi)-silenced lines. However, upon supplementation of the medium with NaCl, the fresh weight (FW) of all lines decreased, with the overexpression lines exhibiting significantly higher FW and lighter coloration compared to the control group (Fig. 6B and C). In contrast, the RNAi-silenced lines showed significantly lower FW (Fig. 6C). Physiological analysis of salt-treated hairy roots revealed that under stress conditions, the overexpression lines accumulated less MDA and more proline (Pro) than the control group. Concurrently, the activities of the antioxidant enzymes CAT, SOD, and POD were significantly elevated in these lines, indicating an enhanced capacity to scavenge ROS and mitigate oxidative damage (Fig. 6D–H). Conversely, the RNAi-silenced lines generally exhibited lower antioxidant enzyme activities and reduced ability to maintain ROS homeostasis, which corresponded with their more severe morphological stress symptoms. The MDA levels in these lines were significantly higher than those in the control under salt stress, suggesting substantial oxidative damage to cellular membranes.

### *SmGA2ox4* overexpression promotes tanshinone accumulation and reduces salvianolic acid content in *S. miltiorrhiza* hairy roots

**Figure 7:**
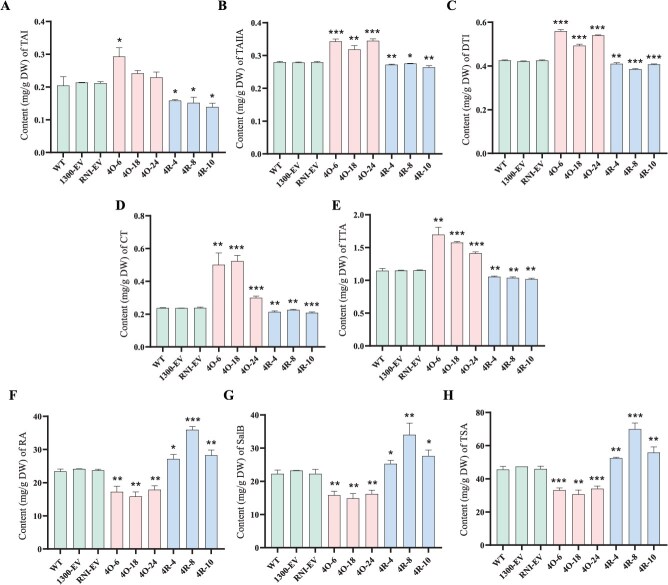
Quantification of tanshinone and salvianolic acid contents in *SmGA2ox4* transgenic hairy roots by HPLC. **(A–E)** Contents of tanshinones in SmGA2ox4-overexpressing and RNAi-silenced hairy roots: **(A)** tanshinone I (TAI), **(B)** tanshinone IIA (TAIIA), **(C)** dihydrotanshinone I (DTI), **(D)** CT, and **(E)** TTA. **(F–H)** Contents of salvianolic acids in SmGA2ox4-overexpressing and RNAi-silenced hairy roots: **(F)** RA, **(G)** Sal B and **(H)** TSA. Data represent mean ± SD of three biological replicates. Significant differences between transgenic lines and corresponding controls were determined by Student’s *t*-test (^*^P < 0.05, ^**^P < 0.01, ^***^P < 0.001).

To investigate the role of *SmGA2ox4* in the biosynthesis of medicinal compounds, we quantified tanshinone and salvianolic acid contents in transgenic hairy roots using high-performance liquid chromatography (HPLC). The empty vector control did not significantly affect the accumulation of either metabolite class. Overexpression of *SmGA2ox4* significantly increased tanshinone content, whereas RNAi-mediated silencing of *SmGA2ox4* led to a notable reduction. Specifically, line 4O-2 exhibited the highest total tanshinone content (1.70 mg/g dry weight (DW)), representing a 1.48-fold increase compared to the WT ATCC line (1.18 mg/g DW). In contrast, line 4R-6 showed the lowest tanshinone accumulation (1.01 mg/g DW), corresponding to a 22% decrease relative to ATCC (Fig. 7A–E).

*SmGA2ox4* exerted an opposite effect on salvianolic acid biosynthesis. While WT hairy roots accumulated 45.66 mg/g DW of total salvianolic acids, overexpression line 4O-18 displayed the lowest content (30.72 mg/g DW), equivalent to 63% of the WT level. In contrast, RNAi-silenced line 4R-8 showed the highest salvianolic acid accumulation (69.97 mg/g DW), reflecting a 1.53-fold increase over the WT (Fig. 7F–H).

These results demonstrate that *SmGA2ox4* functions as a bidirectional regulator, promoting tanshinone biosynthesis while repressing salvianolic acid accumulation in *S. miltiorrhiza* hairy roots.

To elucidate the molecular mechanism by which *SmGA2ox4* modulates the accumulation of tanshinones and salvianolic acids, we analyzed the expression of key structural genes in the respective biosynthetic pathways.

**Figure 8:**
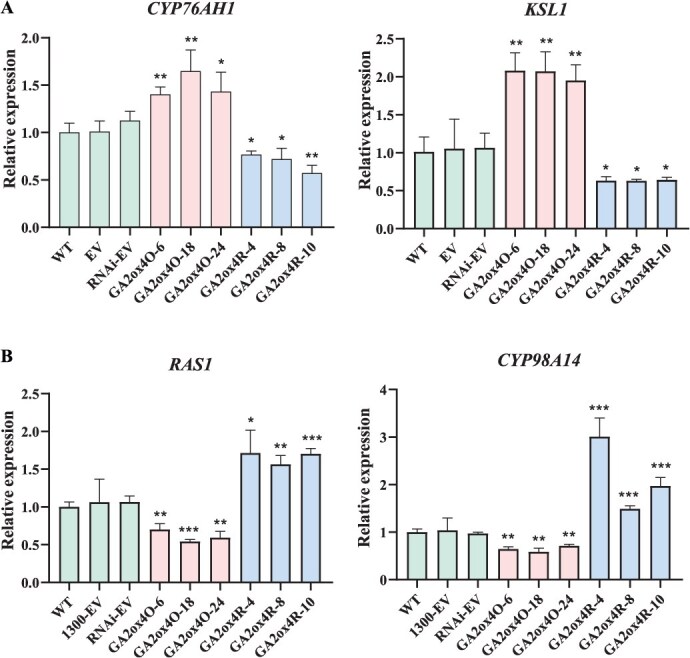
Expression of key biosynthetic genes in tanshinone and salvianolic acid pathways in *SmGA2ox4* transgenic hairy roots. **(A)** Expression levels of tanshinone biosynthetic genes (*SmCYP76AH1* and *SmKSL1*) in *SmGA2ox4*-overexpressing and RNAi-silenced hairy roots. **(B)** Expression levels of salvianolic acid biosynthetic genes (*SmRAS1* and *SmCYP98A14*) in *SmGA2ox4*-overexpressing and RNAi-silenced hairy roots. Gene expression was analyzed by qRT-PCR. Data represent mean ± SD of three biological replicates. Expression levels were normalized to *SmActin*. Significant differences between transgenic lines and the WT control were determined by Student’s *t*-test (^*^P < 0.05, ^**^P < 0.01, ^***^P < 0.001).

In the tanshinone pathway, the expression levels of *SmCYP76AH1* and *SmKSL1* were significantly upregulated in three independent *SmGA2ox4*-overexpressing lines, whereas they were markedly downregulated in three *SmGA2ox4*-RNAi-silenced lines compared to the WT (Fig. 8A). Conversely, in the salvianolic acid pathway, the expression of two key genes—*SmRAS1* and *SmCYP98A14*—was significantly reduced in the *SmGA2ox4*-overexpressing lines, dropping to 54%–70% of the WT levels. In contrast, these genes were substantially upregulated in the *SmGA2ox4*-silenced lines, with the highest-expressing line (4R-4) showing a 1.72-fold increase relative to the control (Fig. 8B).

These transcriptional changes align with the metabolite accumulation patterns, suggesting that *SmGA2ox4* regulates tanshinone and salvianolic acid biosynthesis at the transcriptional level by differentially modulating the expression of pathway-specific key enzyme genes.

## Discussion

### *SmGA2ox4* positively regulates salt stress tolerance and tanshinone biosynthesis in *S. miltiorrhiza*

Accumulating evidence indicates a profound connection between plant secondary metabolism and adaptive responses to environmental stresses [30]. Alterations in environmental conditions can modulate metabolic flux, thereby inhibiting or promoting the biosynthesis of specific secondary metabolites. Plant secondary metabolites (PSMs) fulfill multifaceted roles in defense, pollinator attraction, signaling, and abiotic stress adaptation (reference) [31]. Consequently, shifts in secondary metabolic activity are considered a strategic component of plant environmental adaptation.

Under high salinity, plants experience osmotic stress, ion toxicity, oxidative damage, and nutritional imbalance, collectively impairing germination, seedling growth, and ultimately crop yield [32–34]. Notably, several transcription factors involved in tanshinone biosynthesis in *S. miltiorrhiza* have also been implicated in salt tolerance regulation [35–37]. For instance, *SmMYC2* enhances salt tolerance at the transcriptional level [5], while overexpression of the microRNA *Sm-miR408* reduces ROS accumulation and improves salt tolerance in transgenic tobacco [38].

In this study, we demonstrated that overexpression of *SmGA2ox4* in both *A. thaliana* and *S. miltiorrhiza* hairy roots significantly enhanced multiple physiological indicators of salt tolerance, including increased relative water content, chlorophyll and proline levels, and elevated activities of SOD, POD, and CAT enzymes, alongside reduced MDA content and sodium ion uptake. Concurrently, *SmGA2ox4* upregulates key genes in the tanshinone biosynthetic pathway, leading to elevated accumulation of various antioxidant tanshinones.

These findings provide preliminary evidence that *SmGA2ox4* functions as a dual-regulatory node, coordinating tanshinone biosynthesis with the activation of antioxidant systems to enhance ROS scavenging efficiency and confer stronger salt stress resistance in *S. miltiorrhiza*. This work offers novel insights into improving both stress resilience and medicinal compound yield in this economically important species.

### *SmGA2ox4* acts as a bidirectional regulator of tanshinone and phenolic acid biosynthesis

HPLC analysis revealed that overexpression of *SmGA2ox4* in hairy roots significantly increased the contents of cryptotanshinone, dihydrotanshinone, and tanshinone IIA, whereas RNAi-mediated silencing of *SmGA2ox4* led to a marked reduction in these tanshinones. In contrast, total salvianolic acid content was decreased in the overexpression lines and substantially elevated in the silenced lines, indicating an opposite regulatory effect on the two major classes of bioactive compounds in *S. miltiorrhiza*. Recent advances in the genomics of *S. miltiorrhiza*, including the sequencing of its nuclear, mitochondrial, and chloroplast genomes, have enabled comprehensive elucidation of the tanshinone and salvianolic acid biosynthetic pathways [39, 40]. Key enzyme genes have been cloned and functionally characterized, facilitating metabolic engineering efforts to enhance the production of these compounds [41]. Furthermore, a complex transcriptional network involving multiple transcription factors coordinates their biosynthesis [6]. Positive regulators such as *SmMYC2a*, *SmMYC2b*, and *SmMYB98*, as well as negative regulators like *SmbHLH3*, have been identified. Several factors, including *SmMYB36*, *SmERF1L1*, *SmERF115*, *SmGRAS1*, *SmGRAS2*, and *SmbZIP1*, exhibit bidirectional regulatory roles [42–50].

Among these, *SmMYB36* has been shown to promote tanshinone biosynthesis by directly activating *SmDXS2*, *SmCPS1*, and *SmGGPPS1*, and indirectly via *SmERF6*. Concurrently, it represses salvianolic acid synthesis by downregulating *SmGAPC* (reducing phenylalanine/tyrosine supply) and directly suppressing *SmRAS* expression, as well as indirectly through inhibition of the phenolic acid activator *SmERF115* [45]. Whether *SmMYB36* acts through additional target genes remains to be fully elucidated. Notably, we identified five MYB-binding sites (MBSs) in the promoter region of *SmGA2ox4*, suggesting its potential regulation by *SmMYB36*. This interaction was preliminarily confirmed by dual-luciferase reporter assays (Fig. S3). Although our dual-luciferase assays suggest that *SmMYB36* may transcriptionally activate *SmGA2ox4*, this finding is preliminary and requires further validation through direct-binding assays such as Chromatin Immunoprecipitation PCR or Electrophoretic Mobility Shift Assay. If confirmed, this regulatory link would help explain the coordinated roles of *SmMYB36* and *SmGA2ox4* in balancing stress adaptation and secondary metabolism in *S. miltiorrhiza*. Future studies should focus on experimentally verifying this interaction and elucidating its functional significance in GA-mediated stress and metabolic responses.

### *SmGA2ox4* links the JA signal and GA signal in *S. miltiorrhiza*

Our findings indicate that *SmGA2ox4* responds concurrently to JA and GA induction. JA functions as a critical plant growth regulator involved in diverse processes, including development, stress responses, and secondary metabolite biosynthesis. The role of JA signaling in plant salt tolerance has been well established; for instance, overexpression of *GsJAZ2*, *GaJAZ2*, and *PnJZ1* enhances salt stress resistance [53–55]. GA promotes organ growth by stimulating cell elongation and division, and regulates key developmental transitions such as seed dormancy, germination, vegetative growth, and flowering [51, 52]. As a key GA-inactivating enzyme, GA2ox catalyzes the hydroxylation of bioactive GAs (GA₁ and GA₄) and their precursors (GA_9_ and GA₂₀), converting them into inactive forms such as GA_8_, GA₃₄, GA_51_, and GA_29_. This enzymatic activity enables GA2ox to balance growth and defense under stress conditions. Previous studies have shown that DELLA proteins, repressors of GA signaling, can modulate JA signaling by competitively interacting with JAZ proteins, inhibitors of JA signaling [53]. DELLAs interfere with JAZ1–MYC2 interaction, thereby enhancing MYC2-mediated transcriptional activation. Under high-GA conditions, GA promotes DELLA degradation, allowing JAZ1 to bind and inhibit MYC2. Based on these insights, we propose a regulatory model in which JA signaling may downregulate GA signaling through the induction of GA2ox expression. This hypothesis contributes to a deeper understanding of the complex crosstalk between hormone signaling networks in plants.

To further explore whether *SmGA2ox4* responds to other phytohormonal signals, we treated WT *S. miltiorrhiza* hairy roots with SA, ABA, and indole-3-acetic acid (IAA), and monitored *SmGA2ox4* expression via qPCR (Fig. S4). Interestingly, *SmGA2ox4* expression was significantly downregulated under IAA treatment, while it remained unchanged in response to SA and ABA. This differential hormone responsiveness suggests that *SmGA2ox4* is selectively integrated into specific hormonal pathways—particularly those involving GA and IAA—rather than serving as a general stress-hormone hub. The repression by IAA highlights a potential crosstalk between auxin and GA signaling in modulating stress adaptation and secondary metabolism in *S. miltiorrhiza*. These findings provide a broader perspective on the hormonal network that fine-tunes *SmGA2ox4*-mediated regulation of salt tolerance and metabolite biosynthesis, and underscore the complexity of phytohormone interplay in medicinal plants.

### Potential molecular mechanisms linking *SmGA2ox4* to salt tolerance through GA–DELLA signaling

While our study established a clear functional role for *SmGA2ox4* in enhancing salt tolerance and regulating tanshinone biosynthesis, the direct molecular targets and interaction partners of *SmGA2ox4* remain to be fully elucidated. Based on the well-conserved role of GA2ox enzymes in inactivating bioactive GAs and the established link between GA signaling and stress adaptation, we propose that *SmGA2ox4* influences salt tolerance partly through modulating the GA–DELLA signaling module. DELLA proteins are key negative regulators of GA signaling that accumulate under stress conditions and have been shown to integrate growth inhibition with enhanced stress resilience. In Arabidopsis, salt and cold stress induce the expression of GA2ox genes, leading to reduced bioactive GA levels, subsequent stabilization of DELLA proteins, and activation of antioxidant-related genes such as CAT, Cu/Zn-SOD, and GST^21^. Consistent with this paradigm, our observations of elevated antioxidant enzyme activities (SOD, POD, CAT) and reduced ROS accumulation in *SmGA2ox4-*overexpressing lines suggest that SmGA2ox4 may promote DELLA-mediated antioxidant responses. Moreover, DELLAs have been reported to interact with JAZ proteins, thereby intersecting JA signaling [53]—a pathway that also influences tanshinone biosynthesis and salt tolerance in *S. miltiorrhiza*. We therefore hypothesize that *SmGA2ox4* may stabilize DELLA proteins through the reduction of endogenous GA levels, thereby coordinating ROS scavenging and metabolic reprogramming under salt stress. To validate this model and identify direct interactors or downstream targets of *SmGA2ox4*, future studies should integrate multi-omics analysis, protein-interaction assays such as co-immunoprecipitation, yeast two-hybrid screening, and genetic characterization of DELLA mutants in *S. miltiorrhiza.*

### Conclusion

In summary, this study systematically identified 12 *GA2ox* family genes in *Salvia miltiorrhiza* and functionally characterized *SmGA2ox4* as a key regulator integrating salt stress tolerance and secondary metabolism. *SmGA2ox4* expression is rapidly induced by salt, GA_3_, and MeJA treatments. Overexpression of *SmGA2ox4* in both *Arabidopsis thaliana* and *S. miltiorrhiza* hairy roots significantly enhanced salt tolerance, as evidenced by improved germination, root growth, antioxidant enzyme activities, ion homeostasis, and reduced oxidative damage. Notably, *SmGA2ox4* exerts opposite effects on the two major classes of bioactive compounds—promoting tanshinone accumulation while suppressing salvianolic acid biosynthesis—by differentially regulating key pathway genes such as *SmCYP76A*, *SmKSL1*, *SmRAS*, and *SmCYP98A*. These findings establish *SmGA2ox4* as a dual-functional regulator that simultaneously enhances stress resistance and modulates medicinal metabolite production, providing a promising genetic target for breeding stress-resilient, high-quality *S. miltiorrhiza* varieties.

## Materials and methods

### Genome-wide identification and characterization of GA2ox genes in *S. miltiorrhiza*

The GA2ox family proteins in *S. miltiorrhiza* were identified using a combined bioinformatics approach. First, the known GA2ox protein sequences of *A. thaliana* were retrieved from the NCBI database. These sequences were used as queries to perform a local BLAST search against the *S. miltiorrhiza* genome GCF_028751815.1 (IMPLAD_Smil_shh, RS_2023_06) using TBtools software, with E-value ≤0.01 and a score threshold of >100 to obtain preliminary candidate proteins. Second, the hidden Markov model (HMM) profile of the GA2ox domain (PF03131) was downloaded from the Pfam database. The *S. miltiorrhiza* genomic dataset was subsequently scanned using the HMM Search function in TBtools with an E-value threshold of 1 × 10^−5^ to identify additional candidate sequences.

The candidate amino acid sequences obtained from both methods were then subjected to conserved domain validation. These sequences were submitted to the NCBI Conserved Domains Database (CDD), the Pfam database, and the SMART database to predict their domain architectures. Only those candidates possessing a complete GA2ox domain were ultimately confirmed as members of the *S. miltiorrhiza* GA2ox family.

### Analysis of gene structures and conserved motifs of SmGA2ox genes

The identified genes were systematically renamed according to their homologs in the *S. miltiorrhiza* GA2ox family. The physicochemical properties of each corresponding protein were predicted using the ExPASy online server, and subcellular localizations were inferred with Cell-PLoc 2.0. Conserved motifs within the *S. miltiorrhiza* GA2ox proteins were identified using the MEME suite, with the maximum number of motifs set to six, motif length constrained to 6–50 amino acids, and all other parameters maintained at their default values. The exon–intron structures of the SmGA2ox genes were extracted from the genome annotation file of *S. miltiorrhiza* and visualized using TBtools software.

Furthermore, the 2000-bp genomic sequences upstream of the transcription start sites of the SmGA2ox genes were extracted using the ‘GXF Sequences Extract’ function in TBtools. These promoter sequences were subsequently submitted to the PlantCARE database for *in silico* identification of *cis*-acting regulatory elements.

### Plant genetic transformation

Total genomic DNA was extracted from fresh leaves of wild-type *S. miltiorrhiza* using the 5fz Genome DNA AutoExtraction Kit-DeepWell Plate (Magnetic Beads) on a Nuoto® AutoExtracter-32 Nucleic Acid Extractor (KANGMA-HEALTHCODE (SHANGHAI) BIOTECH CO., LTD, Shanghai, China). To generate the *SmGA2ox4* overexpression transgenic line, the full-length coding sequence (CDS) of *SmGA2ox4* was cloned into the pCAMBIA1300-221-myc vector via homologous recombination. For RNAi-mediated gene silencing, a specific 279-bp fragment of *SmGA2ox4* was inserted into the pCAMBIA1300-pHANNIBAL vector.

For hairy root induction in *S. miltiorrhiza*, the resulting recombinant vectors were introduced into *Agrobacterium rhizogenes* strain ATCC 15834. Subsequently, healthy leaves of *S. miltiorrhiza* were infected with the transformed *A. rhizogenes* to obtain transgenic hairy roots.

Transgenic *A. thaliana* plants were generated via the floral dip method, using *A. tumefaciens* strain GV3101 for transformation.

Following transformation, putative transgenic lines were initially screened by PCR. Positive lines were further subjected to quantitative real-time PCR (qRT-PCR) analysis to determine the expression levels of the target gene, enabling the selection of appropriate transgenic lines for subsequent functional studies.

### Plant hormone and salt stress treatments

Following a 21-day synchronization period, the transgenic hairy roots of *S. miltiorrhiza* were subjected to separate treatments with 150 mM NaCl, 100 μM gibberellic acid (GA₃), or 100 μM MeJA [29]. A control group was established by adding an equivalent volume of anhydrous ethanol. Root samples were collected at 0, 1, 6, 12, and 24 h post-treatment. The expression levels of the target gene were subsequently analyzed using qRT-PCR.

### Quantitative real-time PCR analysis

Total RNA was extracted from root, stem, leaf, and flower tissues using the PrimeScript Kit II. Subsequently, cDNA was synthesized by reverse transcription following the instructions of the Evo M-MLV Reverse Transcription Kit. The resulting cDNA was diluted to a final concentration of 100 ng/μl for subsequent analysis.

qRT-PCR reactions were performed using the ChamQ SYBR qPCR Master Mix. Each 15 μl reaction mixture contained 1 μl of cDNA template, 1 μl each of forward and reverse primers, 7.5 μl of 2× ChamQ SYBR qPCR Master Mix, and 4.5 μl of ddH₂O. The amplification program consisted of an initial denaturation at 95°C for 30 s, followed by 40 cycles of denaturation at 95°C for 10 s and annealing/extension at 60°C for 30 s. A melt curve analysis was subsequently conducted by heating to 95°C for 15 s, cooling to 60°C for 60 s, and then gradually increasing to 95°C.

The Actin2 gene of *S. miltiorrhiza* was used as an internal reference for normalization. Relative gene expression levels were calculated using the 2^−ΔΔCT^ method. All samples were analyzed with three technical replicates.

### Salt stress tolerance assays in *A. thaliana*

#### Seed germination assay

Seeds of WT and two independent transgenic *A. thaliana* lines were surface-sterilized and uniformly sown on one-half MS medium supplemented with 0, 150, 200, or 250 mM NaCl [28, 29]. The germination rate was monitored and recorded daily over a period of 8 days.

Salt stress tolerance assays: the selection of 150–250 mM NaCl for *A. thaliana* was based on preliminary dose–response experiments conducted in our laboratory (data not shown). For *A. thaliana*, concentrations below 100 mM did not induce clear phenotypic differences, while 300 mM NaCl severely inhibited germination and growth. Therefore, 150–250 mM NaCl was selected to ensure measurable yet nonlethal stress responses.

#### Root length assay

After 3 days of germination on standard one-half MS medium, uniformly grown WT and transgenic seedlings were transferred to one-half MS medium containing 0, 150, 200, or 250 mM NaCl. The plates were positioned vertically to allow for clear root growth direction. The length of the primary roots was measured and recorded on the eighth day post-transfer.

#### Phenotypic and physiological analysis of seedlings

Four-week-old soil-grown WT and transgenic plants were irrigated with 250 mM NaCl solution every 3 days for a duration of 2 weeks. Control plants were watered with an equal volume of distilled water. Phenotypes were documented on the 7th and 14th days of treatment [54]. For physiological assays, aerial parts of plants treated for 14 days were harvested. Tissues were homogenized in ice-cold phosphate buffer (50 mM, pH 7.8) and centrifuged at 12 000 × g for 15 min at 4°C. The resulting supernatant was collected as the crude enzyme extract for subsequent biochemical analyses [55].

#### Malondialdehyde content

A 20-μl aliquot of the enzyme extract was added to 2 ml of 0.6% thiobarbituric acid (TBA) solution. The mixture was heated in a boiling water bath for 15 min, cooled, and centrifuged. The absorbance of the supernatant was measured at 450, 532, and 600 nm. MDA concentration was calculated using the extinction coefficient 155 mM^−1^ cm^−159^.

#### Proline content

Proline content was quantified using the acid–ninhydrin method [56].

#### Superoxide dismutase activity

SOD activity was determined by adding 20 μl of enzyme extract to 3 ml of reaction mixture containing phosphate buffer (50 mM, pH 7.8), methionine, NBT, EDTA, and riboflavin. The reaction was initiated by exposing the mixture to 4000 Lux of light for 30 min, and absorbance was measured at 560 nm. One unit of SOD activity was defined as the amount of enzyme required to inhibit 50% of NBT reduction.

#### Peroxidase activity

POD activity was assayed by adding 20 μl of enzyme extract to 3 ml of reaction solution containing phosphate buffer (50 mM, pH 7.8), guaiacol, and H₂O₂. The increase in absorbance at 470 nm was recorded every minute. Enzyme activity was calculated using the extinction coefficient 26.6 mM^−1^ cm^−1^.

#### Catalase activity

CAT activity was measured by adding 0.1 ml of enzyme extract to 2.5 ml of phosphate buffer (50 mM, pH 7.0) containing H₂O₂. The decrease in absorbance at 240 nm was recorded every minute. Enzyme activity was calculated using the extinction coefficient 39.4 mM^−1^ cm^−1^.

#### Chlorophyll content

Fresh leaf tissue was homogenized in 80% acetone and centrifuged. The absorbance of the supernatant was measured at 663 and 645 nm. Chlorophyll content was calculated using Arnon’s equations.

#### Leaf relative water content

Fresh leaves were weighed to obtain FW, immersed in distilled water for 6 h to obtain turgid weight (TW), and then dried at 80°C to constant weight for DW. RWC was calculated as: RWC (%) =[(FW – DW) / (TW – DW)] × 100 [57].

#### Sodium and potassium ion content

Whole plants were dried at 80°C to constant weight, ground to a fine powder, and digested in 1 M HCl. Sodium and potassium ion concentrations in the digest were determined using a flame spectrophotometer.

### Phenotypic, physiological, and biochemical analyses of *S. miltiorrhiza* hairy roots

WT and two independent transgenic hairy root lines of *S. miltiorrhiza* (0.3 g each) were inoculated into 100-ml conical flasks and cultured under identical conditions for 21 days to achieve synchronized growth. The hairy roots were then transferred to fresh medium supplemented with 150 mM NaCl and subjected to salt stress for 7 days. After treatment, the hairy roots were harvested, photographed, and their FW was recorded.

Salt stress tolerance assays: The selection of 150 mM NaCl for *S. miltiorrhiza* hairy roots was based on preliminary dose–response experiments conducted in our laboratory (data not shown). For *S. miltiorrhiza* hairy roots, 150 mM NaCl was found to significantly affect growth and physiological responses without causing lethality.

Subsequent physiological and biochemical analyses—including measurements of MDA, proline, antioxidant enzyme activities (SOD, POD, CAT)—were performed following the same methodologies as described for the *A. thaliana* experiments.

### Quantification of gibberellin content in *S. miltiorrhiza* hairy roots

The endogenous GA₁ content in transgenic hairy roots was determined using a commercial enzyme-linked immunosorbent assay (ELISA) kit (ml077221, Mlbio, China) according to the manufacturer’s protocol. Briefly, ~0.2 g of fresh hairy root tissue was homogenized in 8 ml of phosphate-buffered saline (PBS, pH 7.4). The homogenate was centrifuged at 10 000 × g for 15 min at 4°C, and the supernatant was collected for analysis. The extracted samples were then subjected to the ELISA procedure, which included antigen–antibody binding, enzyme–conjugate reaction, and color development with the substrate solution. Absorbance was measured at 450 nm using a microplate reader, and GA₁ concentrations were calculated based on a standard curve run in parallel. Each measurement was performed with three biological replicates.

### Quantification of tanshinones and phenolic acids in hairy roots by high-performance liquid chromatography

Lyophilized transgenic and WT hairy roots were pulverized into a fine powder and subjected to methanol extraction. HPLC analysis was performed following an established protocol (citation). The chromatographic separation was achieved using a binary gradient elution system at a flow rate of 1 ml/min, with the column temperature maintained at 35°C and an injection volume of 10 μl.

Tanshinone compounds were detected at a wavelength of 270 nm, while phenolic acids were quantified at 288 nm. Total phenolic acid (TPA) content was calculated as the sum of salvianolic acid B (Sal B) and rosmarinic acid (RA). Total tanshinone (TTA) content was determined as the sum of cryptotanshinone (CT), tanshinone IIA (TAIIA), tanshinone I (TAI), and dihydrotanshinone I (DTI).

### Statistical analysis

All data were analyzed using SPSS statistical software (version 24.0). Student’s *t*-test and one-way analysis of variance (ANOVA) were employed for comparisons between two groups and multiple groups, respectively. Each experiment was conducted with three independent replicates, and data are presented as mean ± standard error (SE). Significance levels were denoted as follows: ^*^P < 0.05, ^**^P < 0.01, and ^***^P < 0.001.

## Supplementary Material

Web_Material_uhag058

## Data Availability

The datasets supporting the results of this article are included within the article and its supplementary file.
