# Supplementary material for: SmGA2ox4 plays a positive role in improving the salt tolerance and tanshinone accumulation of Salvia miltiorrhiza
Source: Hortic Res. 2026 Feb 26;13(6):uhag058. doi: 10.1093/hr/uhag058 (PMC13246269; doi:10.1093/hr/uhag058)
Supplement: Web_Material_uhag058 [file web_material_uhag058.zip › Supplementary figure - R1.docx]

**Supplementary Figure 1. Identification and Validation of *SmGA2ox4*-Transgenic *A. thaliana* Lines**

**(A)** PCR-based screening of transgenic *A. thaliana* plants. Lanes: M, 2000 bp DNA marker; N, no-template negative control; P, positive control (pCAMBIA1300‑221‑myc‑*SmGA2ox4* plasmid); WT, wild‑type A. thaliana; remaining lanes represent independent transgenic lines. **(B)** qRT‑PCR analysis of *SmGA2ox4* expression in transgenic lines. Transcript levels were normalized to *AtActin*, with expression in line OE‑2 set as 1. Data are presented as mean ± SD of three biological replicates. Significant differences relative to the wild‑type control were determined by Student’s *t*-test (**P* < 0.05, ****P* < 0.001).

**Supplementary Figure 2. qRT‑PCR analysis of *SmGA2ox4* expression in overexpression and silenced hairy root lines of *S. miltiorrhiza*.**

**(A)** Transcript levels of *SmGA2ox4* in 16 independent overexpression lines. Lines 4O‑6, 4O‑18, and 4O‑24 exhibited the highest expression. **(B)** Transcript levels of *SmGA2ox4* in 16 independent RNAi‑silenced lines. Lines 4R‑4, 4R‑8, and 4R‑10 showed the lowest expression. Gene expression levels were normalized to *SmActin*, with the expression level in ATCC set as 1. Data represent mean ± SD of three biological replicates. Significant differences between treatment groups and the control were determined by Student's *t*-test (**P* < 0.05, ***P* < 0.01, ****P* < 0.001).****

**Supplementary Figure 3. Analysis of the regulatory interaction between *SmMYB36* and *SmGA2ox4*.**

**(A)** Overexpression of *SmMYB36* upregulated *SmGA2ox4* transcript levels in *S. miltiorrhiza* hairy roots. Gene expression levels were normalized to *SmActin*, with the expression level in ATCC set as 1. Data represent mean ± SD of three biological replicates. Significant differences between treatment groups and the control were determined by Student's *t*-test (***P* < 0.01, ****P* < 0.001). **(B‑C)** Transcriptional activation of the *SmGA2ox4* promoter (pro*SmGA2ox4*) by *SmMYB36*. **(D)** Dual‑luciferase (LUC) assay of truncated pro*SmGA2ox4* promoter segments. Based on the presence of five MYB‑binding sites (MBS), the promoter was divided into three fragments: P1 (positions 1–250), P2 (1–500), and P3 (250–500).

**Supplementary Figure 4 Expression patterns of *SmGA2ox4* in response to SA, ABA, and IAA treatments.**

Temporal expression patterns of *SmGA2ox4* in hairy roots following 24-hour treatments with **(A)** 50 μM SA, **(B)** 10 μM ABA, and **(C)** 10 μM IAA. Expression levels were normalized to SmActin and are presented relative to the 0-hour control group (set as 1). Data represent mean ± SD of three biological replicates. Significant differences between treatment groups and the control were determined by Student's *t*-test (***P* < 0.01).
