# Supplementary material for: SmGA2ox4 plays a positive role in improving the salt tolerance and tanshinone accumulation of Salvia miltiorrhiza
Source: Hortic Res. 2026 Feb 26;13(6):uhag058. doi: 10.1093/hr/uhag058 (PMC13246269; doi:10.1093/hr/uhag058)
Supplement: Web_Material_uhag058 [file web_material_uhag058.zip › Supplementary Table 1.docx]

**All Identified SmGA2ox CDS Sequences**

**SmGA2ox1**

ATGGAGAATATTACTGTAGGAGATGATCATATAGAGCTCCCAATCGTAGACCTTAGGAGGGAGCGATCAGAAGCTTCGAGAATGATCGTAAAAGCGTGCGAGGAGTTCGGATTCTTCAAGGTGATAAACCATGGTGTGAAGCAGGAGATTATTTCTCTCATGGAAGAAGAAGCTTCAATGTTTTTCTCCAAACCCGTATCCGAGAAGAACCGGGCCGGACCTCCGGATCCCTACGGATATGGTTCCCACAATATTGGGTTAAACGGCGACGTTGGTGAGGTTGAGTATCTTCTCCTCCAAGCCAACCCTCTTTTCATCTCTCACAAATCTAAATATATCTCTTCCCATCCCCAACAATTCAGCTACGTAGTGAGCAGGTATGTGGAAGCTGTGAGGGAGTTGGCCTGTGAGATATTGGATCTGATCGTAGAGGGATTATGGGCTGAGGCCCAGCCCGTTTCAACGCTTAGTGGGCTTGTTCGTGATCTCGACAGCGATTCCCTCATCAGGCTCAATCACTACCCGCCCACTGACGTCAGCCAGCCATCGGCCCACAAGATCGGGTTCGGGGCTCATACCGACCCTCAGATCCTTACAATCCTCAGATCCAACGGCTCCAGCGGCCTCCAACTCTCATTGCAGGGTGGGGTGTGGGTCCCCGTTGACCCACACCCGCCCTCCGCTTTTTGCGTTAACGTCGGTGACGTCTTGCAGGCTATGACGAATGGGAGGTTTGTAAGTGTAAAACATAGAGCTGTGGTGAATTCGATAAAGTCGAGAATGTCGATAGTTTATTTTGCTGCGCCGCCGTTGCATGCTACCATAACTTGCATTGCTTCAAATCCTACGTATCGAAGCTTCAGTTGGGGCGAGTACAAGAAAGTTGTCTACACGGGTAGGCTCAACGACTGCCGCCTTAATCTTTTCAAACTCCACTGA

**SmGA2ox2**

ATGGTTGTGATATCTCAAAATCCAACCATGAAAATGGAGAAAATACGAGACGACATTGAGCTTCCAATCATAAACCTCTGCAACCGATCAGAAGCGATGAAAGAGATGGTGAAAGCCTGTGAAGAATATGGTTTCTTCAAAGTGATCAACCATGGCGTGCCACAGGGGATAATTTCTCAGGTGGAAGAAGAAGCCCGCGGCTTCTTCGCCAAGCCCGGGCCCGAAAAGATGCGGGCTGGGCCCCCCTATGGCTGCAAGAACATAGGCTTGCAGGGCGACGTCGGAGAAGTCGAATATCTCATTCTCCAAACCAACTCCCCCTTCATAATTCCTACCGATGAATCCAATAAATTCAGGTCAGCAATAAATGCGTACGTGGAAGCAGTGAGGAAGCTGGCATGTGATATATTGGATCTATTGGTGGAAGGAGTGTGCGGGTCGGAGGGTGGATCGGCGTTGAGTAGGCTAATGAGAGACAGTGAGAATGACTCAATCCTAAGGTTGAATCACTACCCGGCGGGCGACGTGAGCAAGATCGGGTTTGGCGAGCACACTGACCCTCAGATCATCACCCTTCTCCGATCCAACGGTGTGGAGGGCCTCCAGATCTCCGTCCAGGACGGCCTGTGGGTCCCGGTCAACCCCTACCCGGACTCTGCCTTCTGCGTCAATGTGGGCGACATCTTACGGGTAATGACGAATGGAAGGTTTGTGAGCGTGAAGCACAGAGTGGCGGTGCAAGCATACGAATCAAGAATGTCGATTGCGTACTTCGTTGCTCCAGCGCTGCATGCGACGGTGAGCTGCCTTCCGGGGCTGGGGCTGCCGCTCTACAGAAGCTTCACTTGGGGTGAATACAAGCAAGCTGTTTATACAGGCAGGCTAGCCGATACCCGCCTCAATCTCTTTGCATTGCCCTCTCATCACAATAATTAA

**SmGA2ox3**

ATGACATGCGAGATTCTAGAAATGGTGGGTGAAGGCCTGTGGGCCCAGGATAAATCTATTTTTAGTAAGCTTATCGAAGATGGAAGCAGCGATTCCTGCTTCCGGATCAATCACTATCCTTCAGTCAATGCCGCTGCCGACATCGACGACGATGATTGGCGGACTCGCCCCAAACCGTTGGACGACCCGTCCAAGATCCGGATCGGGTTCGGCGAGCATTCGGACCCCCAGATCCTCACCATCCTCAGATCCAACAACGTCGCGGGCCTCCAGATCCTCTCCGGGGACGGGTTGTGGGTCCCCGTCCCGCCCGACCCGCACAACTTCTGCGTCTTCGTCGGCGACGCCTTTCAGGCCCTCACCAACGGCAGATTCACGAGCGTGCGGCACAGGGTGGTGGCGCGCTCGGCCAAGGCGAGGATGTCGATGATGTATTTCGCGGCGCCGCCTCTCGCCGCCACGATCTCGCCCATGCCGGAGCTGCTCTCCGCCCAAAATCCGAGTATTTATAGGGCGTTCACGTGGGGGGAGTATAAGACCACCGCTTATTCATTGAGATTGGCAGATCATCGTCTCGACCTCTTTAGAAAATAA

**SmGA2ox4**

ATGAACTGCATGGGCGATTTAATTGGGGGAAAATCGGATCGGATCGGATTGCAGGGAGATGGTGAGCGAGTACGTAGCGGCGGTGCGAGAGATGGCGTGCTCGGTGCTGGAGATGATCGCGGAGGAGCTGGAGATGGAGGCGCGGGAGGCGCTGAGCCGTCTGATCAGCGACGAGAGGAGCGACTCGTGCTTCCGCGTGAACCACTACCCGCCTGCCCGGAGCTGCAGGCATTGAGCGGTCGGAGCCTGATCGGCTTCGGGGAACACACGGACCCACAAATAATATCCGTTTTGAGGTCCAACAACGCGTCAGGCCTGCAAATCTGCGACGCCGATGCCACGTGGCTCTCTGTCCCACCCGATCACACCACGTTTTTCTTCATCGTTGGGGATTCGCTGCAGGTGATGACTAACGGGAGGTTCCGGAGCGTGAAGCACAGGGTGTTGGCGGACAGCCGGAAATCGCGAGTGTCGATGATATACTTCGGGGGGCCGCCGCTGACGGAGAAGATAACGCCGTTAGCGGGGGTGATGGAGGAAGGGGAGGAGAGTTTGTATAAGGAATTCACGTGGTGTGAATACAAGAAGTCAGCCTACAAGACAAGGTTGGGTGACAATAGGCTAAGCCTTTTTGAGAAGTCTGCTACTGCTGCTGCCACTGCCGCTGCTGCCCAATAG

**SmGA2ox5**

ATGGTGGTGCTATCTCAGCCGATTGTCGATGGCAATCTGGCCCACATCAAGACATGCAAGGCGGCCGCGAACGCCGTCGCCATCCCCGTCGTCGACCTCTCGGAATCCGGCGCCAAAACCGTCATCGCGGACGCCTGCAGAGACCTCGGCTTCTTCAAGGTGATCAACCACAGAATCTCCATTGATTTCCTGGCCGAAATGGAGGCGGAGGCCATGAAGTTCTTCAAACTGGCGCCGCAGGAGAAGGCGCAATTCGGGCCCCCGAACCCCTTCGGCTACGGCAGCAAGGTCATCGGCCCCAACGGCGACGTCGGGTGGGTCGAGTACCTCCTCTTCTCCACCAACCCCGACCTCATCTCCCGCAACCCTAACTTCGCCGCGCCGGGGATCTCCCAAACATTATGGTAA

**SmGA2ox6**

ATGGTTCGGCCGGAATCCTTGAGGCCTCCCAACTCAGCTTCGTCAGCCTTGATCCTTGACGAAAATAATAAAGCCTTCATAAGAACAAATCAAGATAAACATTGCTCTGACATCAAGACATGCAAGGCGGCCGCCAACGCCGTCGCCATCCCCGCCGTCGACCTCTCGGAATCCGGCGCCAAAACCGTCATCGCGGACGCCTGCAGAGACCTCGGCTTCTTCAAGGTGATCAACCACAGAATCTCCATGGATTTCCTGGCCGAAATGGAGGCGGAGGCCATGAAATTCTTCAAACTGGCGCCGCAGGAGAAGGCGCAATTCGGGCTCCCGAACCCCTTTGGCTACGGCAGCAAGGTCATCGGCCCCAACGGCGACGTCGGGTGGGTCGAGTACCTCCTCTTCTCCACCAACCCCGACCTCATCTCCCGCAACCCAAACTTCGCCGCGCCGGGGATCTCCCAAACATTATGGGAGATGGTGAGCGAGTACGTATCGGCGGTGCGAGAGATGGCGTGCTCGGTGCTGGAGATGGAGGCGCGGGAGGCGCTGAGCCGGCTGATCAGCGACGAGAGGAGCGACTCGTGCTTCCGCGTGAACCACTACCCAGCCTGCCCGGAGCTGCAAATCTGCGACGCCGACGCCACGTGGCTCTCTGTCCCACCCGATCACACCACGTTTTTCTTCATCGTTGGGGATTCGTTGCAGGTTCCGGAGCGTGAAGCACAGGGTGTTGGCGGCCCCATTCAAGTTCCATCCGACAACTGGCTATCGTTTAGAGAAGAGAGAGACACCTTTTGTTTCATCTGTTTTTATTACTATTGTGAAGTAACACTTCCAGCGCTCGCTGGAACTCAGCGGTCGCTGGGTTTCCAGCGGGTGCTGGGACTCCGCGGGCGCTGGGTCTCTTCTAGCGGGCGCTGGTTTCATTGA

**SmGA2ox7**

ATGTGGGAACGTGTGCAGAATATGTATCATGAAGCTCAAAAAGAAAATCCAGATGAGATCAGCCCAAGGAATATCGAATCAATGAAAGGTCGTTTTAAACGACTTAATGAAAATGCAAACAAGTGGATTGCTGCTTGCAAAGAAGCAAATGCTAGAAAAAGAAGTGGAATGAGCCAGAAAGATATAGAGATGGAAGCTCACTCAATTTATGATGCAGGTGGCAGTAAATTCCAAGACTTGGTTGTTTTCAATGATGTTATGAGTAAACATCCGAAGTGGAACTTAGCAATCAATGAAGTGGGTGATGATCAAGAAAGTGGTGGCAGCACAAAAAGGTCTAAGACTTCTGAAGATGGTGATTACTTTATCCCGTCCAATCCAGAAACTCCAACAACTGGCGGATCTACTATGTCTCGTCCTACAGGTAGAGACAAAGCTAAAAGGAAAGGAAAAGCGCCCGCGGCCAAGAAGCCAGCGAGCGCTGGAACTCAGCAATCGTTGAGTGTCCAGCGAGCGCTGGATAAGCATTGCTCTGACATCAAGACATGCAAGGCGGCCGCCAACGCCGTCGCCATCCCCGTCGTCGACCTCTCGGAATCCGGCGCCAAAACCGTCATCGCGGACGCCTGCAGAGACCTCGGCTTCTTCAAGGTGATCAACCACAGAATCTCCATGGATTTCCTGGCCGAAATGGAGGCGGAGGCCATGAAATTCTTCAAACTGGCGCCGCAGGAGAAGGCGCAATTCGGGCTCCCGAACCCCTTCGGCTACGGCAGCAAGGTCATCGGCCCCAACGGCGACGTCGGGTGGGTCGAGTACCTCCTCTTCTCCACCAACCCCGACCTCATCTCCCGCAACCCAAACTTCGCCGCGCCGGGGATCTCCCAAACATTATGGGAGATGGTGAGCGAGTACGTATCGGCGGTGCGAGAGATGGCGTGCTCGGTGCTGGAGATGATCGCGGAGGAGCTGGAGATGGAGGCGCAGGAGGCGCTGAGCAGGCTGATCAGCGACGAGAGGAGCGACTCGTGCTTCCGCGTGAACCACTACCCAGCCTGCCCGGAGCTGCAAATCTGCGACGCCGACGCCACGTGGCTCTCTGTCCCACCCGATCACACCATGTTTTTCTTCATCGTTGGGGATTCGTTGCAGGTGATGACTAACGGGAGGTTCCGGAGCGTGAAGCACAGGGTGTTGGCGGCCCCATTCAAGTTCCATTCGACAACTGGCTATCGTTTAGAGAAGAGAGACACCTTTTGTTTCATCTGTTTTTATTACTATTGTGAAGTACCACACTTCCAGCGCTCGCTGGAACTCAGCGGTCGCTGGGTTTCCAGCGGGTGCTGGGACTCCGCGGGCGCTGGGTCTCTCTGGTTTCATTGA

**SmGA2ox8**

CTGGGCGAGTATGTCTCTGCTGTCAGAAACATGTTGACCGGAGTTCTGGACATGATTTCCGAGGAACTGAGAATCGGGCCGACGGATTTCTTGGGCGGGCTGATCGGAGACGAGAAGAGCGATTCGTGTTTCCGGCTGAACCACTACCCGCCGTGCCCGGATCTTGAGGCATTGAGCGGCCGGAATTTGATCGGATTTGGTGAGCACACAGACCCTCAAATAATGTCTGTTTTGAGATCTTCCGACGCGCCGGGCCTCGAAATCTGTCTGAAAAATGGTTCCTGGGCTTCTGTCCCATCTGATCAGACCTCCTTCTACTTCAGCGTTGGAGATTGTCTGCAGGTGATGAGCAATGGAAGATTTAAGAGTGTGAAGCACCGAGTGGTGAGTAACTGCCTGAAATCAAGGCTGTCAATGGTATATTTTGGAGGGCCACCATTGAGTCAAAAGATACGCTCATTAATAATGGAGGAAAGTGAAGAAAGTTTGTACCACGAATTCACTTGGTCTCAATACAAGAAATCAGCTTACAACTCAAAGTTGGGTGATAATAGGTTGAAGCTTTTTGAGAAAAATCAAGTCATGTTTTCTTAA

**SmGA2ox9**

ATGGTACTCCATTCCAAACCAGCAATCGAACAGTTGTCCAAAGCCAATAACTGCGCCGCATTTTTGGGCGGCATCCCGCTCATCGACCTCTCCAAACCTGACTCCAAAGCGCAGCTCGTCCGAGCCTGCGAGGACTTGGGCTTCTTCAAGGTCATCAACCACGGCGTCCCGGCCGACCTCATCAGAGAGCTGGAGTCGGAAGCCGTCAGATTCTTCTCCCTCCCTCTCTCCGTGAAGGAGCAGGCCGGCCCGCCCGACCCCTTCGGCTACGGCGACAAGAAGATCGGCCCCAATGGCGACGTCGGATGGGTCGAATACCTGCTCCTCACCACCAACACCCACAACTTCCCATCCATCTTCGGCGAGGCCGCCGACAAATTCCGCTGGCTGGTGAATGAATACATCTCGGCCGTCAAGAAGATGGCCTGCGAGATTCTGGAAATGTTGGCGGAGGGATTGCGGATTCAGCCCAGGGATGCTTTCAGCAAGCTGCTCATGGATGAACAGAGCGACTCTGTTTTCCGGGTGAATCACTACCCTCCGTGCCCGGACGTTCAGGGACGCAATTTGATCGGCTTCGGCGAGCACACCGACCCTCAGATCATCTCTGTGCTCAGATCCAACAACATTTCCGGCCTTCAAATCTCCCTCAAAGACGGCCGCTGGATTTCAGTCCCCTCCGACCACAACTCCTTCTTCATTAATGTTGGCGACTCCTTACAAGTGATGACTAACGGGAGGTTTAAGAGCGTGAGGCACAGAGTGGTGGCCAACGGTTCGAAATCGAGGCTGTCAATGATTTATTTTGGAGGACCACCATTGAGTGAAAAGATAGCTCCATTGGCATCACTCATGCAAGGGGAAGAAGACAGCTTGTATAAGGAATTTACATGGTTTGAATACAAGAAATCGGCTTATAAGTCAAAATTGGCCGATAATAGACTAGGCCTCTTTGAGAAGATTGCAGCCTCATGA

**SmGA2ox10**

ATGGTGGTTTTATCAAAGGCAGTTGATAGTGACTTCCAAACATGCAAATCCAGCTCCGACATTTTCACCGGAATCCCAGTGGTGGATTTCTCAGATGCCATTAACGCAAAGAAGCTGATCGTGGAAGCCTGCAAAGAATTCGGATTCTTTAAGGTCATCAACCACGGAATCTCAATGGAGGTGGTGGCCATGTTAGAATCCGAGGCTGTCAACTTCTTCGCACTGCCGCAACAAGAAAAGGAGAAGCCGGGCCCTACCAGGCCGTTCGGCTACGGCAACACCAGAATCGGGCCCAACGGCGACACGGGCTGGCTCGAGTACCTCCTCCTCTCCACCGGCCCGCAACCCCATTTCCCCGCCATTTCACAAACCGTGCTGGGCGAGTATGTCTCTGCTGTCAGAAACATGTTGACCGGAGTTCTGGACATGATTTCCGAGGAACTGAGAATCGGGCCGACGGATTTCTTGGGCGGGCTGATCGGAGACGAGAAGAGCGATTCGTGTTTCCGGCTGAACCACTACCCGCCGTGCCCGGATCTTGAGGCATTGAGCGGCCGGAATTTGATCGGATTTGTGAAAGACGTGATGCAGGTGATGAGCAATGGAAGATTTAAGAGTGTGAAGCACCGAGTGGTGAGTAACTGCCTGAAATCAAGGCTGTCAATGGTATATTTTGGAGGGCCACCATTGAGTCAAAAGATACGCTCATTAATAATGGAGGAAAGTGAAGAAAGTTTGTACCACGAATTCACTTGGTCTCAATACAAGAAATCAGCTTACAACTCAAAGTTGGGTGATAATAGGTTGAAGCTTTTTGAGAAAAATCAAGTCATGTTTTCTTAA

**SmGA2ox11**

ATGGTGTTTCTCTCCAAACCGGCAATGGAACAGTTATTGCCGCTGATCGACCTCTCGGAACCCGACTCCCAGTCGCAGCTCGTCAAGGCCTGCGAGGAGGTCGGCTTCTTCAAGGTCATCAACCACGGCGTCCCCTTCCAATTCATGGCCGACTTGGAATCCGAAGCCATCAAATTCTTCTCCCTCCCCTTATCGGATAAGGAGAAAGCCGGCCCCCCGGACCCCTTCGGCTACGGACACAAGAACATCGGCTGCACCGGCGATGTCGGCTGGGTCGAATACCTCCTCTTTACCACCGACGCCGATTCAATCTACCACAAGTTCGCCTCCATTTTCGGCGAGGCTGCCGACAGATTCCGTTGCGTTTTGAATGAGTACTTATCGGCGGTGAAGAAGATGGCATGCCGGATTCTAGAGATGGTGGCGGAGGGGCTGAAGATTCAACCGCGGGATGTGTTGAGCAAGCTGCTGATGGACGAGCAGAGCGACTCTGTTTTCCGGGTGAACCACTACCCGGCGCGGCCGGAGACTGAGGGTTTGATCGGATTCGGAGAGCACACGGATCCGCAGATAATATCGGTGCTGAGATCGAACAACACGTCGGGGCTGCAAATATCTTTGAAGGAAGGGGATTGGATTTCCATCCCGCCAGATCAATATTCCTTCTTCATTAATGTGGGTGACTCCTTGCAGGTACAACACTTTCGCGCCGTCAATTCTCTCTCATAA

**SmGA2ox12**

CTGCGAGGACTTGGGCTTCTTCAAGGTCATCAACCACGGCGTCCCGCCGACCTCATCAGAGAGCTGGAGTCGGAAGCCGTCAGATTCTTCTCCCTCCCTCTCTCCGTGAAGGAGCAGGCCGGCCCGCCCGACCCCTTCGGCTACGGCGACAAGAAGATCGGCCCCAATGGCGACGTCGGATGGGTCGAATACCTGCTCCTCACCACCAACACCCACAACTTCCCATCCATCTTCGGCGAGGCCGCCGACAAATTCCGCTGGCTGGTGAATGAATACATCTCGGCCGTCAAGAAGATGGCCTGCGAGATTCTGGAAATGTTGGCGGAGGGATTGCGGATTCAGCCCAGGGATGCTTTCAGCAAGCTGCTCATGGATGAACAGAGCGACTCTGTTTTCCGGGTGAATCACTACCCTCCGTGCCCGGACGTTCAGGGCCGCAATTTGATCGGCTTCGGCGAGCACACCGACCCTCAGATCATCTCTGTGCTCAGATCCAACAACATTTCCGGCCTTCAAATCTCCCTCAAAGACGGCCGCTGGATTTCAGTCCCCTCCGACCACAACTCCTTCTTCATTAATGTTGGCGACTCCTTACAAGTGATGACTAACGGGAGGTTTAAGAGCGTGAGGCACAGAGTGGTGGCCAACGGTTCGAAATCGAGGCTGTCAATGATTTATTTTGGAGGACCACCATTGAGTGAAAAGATAGCTCCATTGGCATCACTCATGCAAGGGGAAGAAGACAGCTTGTATAAGGAATTTACATGGTTTGAATACAAGAAATCGGCTTATAAGTCAAAATTGGCCGATAATAGATTAGGCCTCTTTGAGAAGATTGCAGCCTCATGA
